# Effect of globalization on global dental caries trend

**DOI:** 10.1097/MD.0000000000021767

**Published:** 2020-08-28

**Authors:** Bakr Salem Alsuraim, Dong-Hun Han

**Affiliations:** Department of Preventive and Social Dentistry, School of Dentistry & Dental Research Institute, Seoul National University, South Korea.

**Keywords:** caries, epidemiology, global health, public health policy

## Abstract

Risk factors such as smoking and sugar intake threaten the health of human being at an individual national level as well as at a global level. The globalization affect health indirectly through macro and micro-level factors. This study aimed to identify the global trend of dental caries according to countries national income level, and to examine the role of globalization, health services, obesity, and sugar consumption on dental caries. Data for 160 countries were collected for the time period of the 1990s to 2010s. The final sample included 46 countries with complete data (21 high income countries (HIC) and 25 middle and low income countries (MLIC)). The main dependent variable was the mean decayed, missing, and filled teeth (DMFT) index of 12-year-olds as an indicator of dental caries. Globalization was a main independent variable which was measured by economic growth, urbanization and economic freedom. Other independent variables were health services, obesity and sugar consumption. The data were analyzed first using repeated measures analysis of variance to compare dental caries trends in HIC and MLIC. Then, using multiple linear regression and partial least squares structural equation modeling (PLS-SEM), the relationships between globalization, health services, obesity, sugar consumption, and dental caries were examined. The results of PLS-SEM revealed that globalization was associated with lower DMFT in HIC. The global dental caries trend had a declined pattern, but this pattern has been attenuated in MLIC after the new millennium. There is a need for policy change and regulations on sugar trade especially in MLIC to diminish the adverse consequences of globalization, and to improve population dental health.

## 1 Introduction

Dental caries is considered to be the most common chronic disease in industrial and in most lower income countries.^[1]^ In Global Burden of Disease 2010 Study, 3.9 billion people were affected by oral diseases, and untreated caries in permanent teeth were assessed as the most common disease with a worldwide prevalence of 35%.^[2]^

Narrative reviews described a clear decrease of caries prevalence over decades in developed countries.^[3]^ In the developing countries, a review has reported a global increase in the prevalence of caries.^[4]^ However, other systematic reviews have reported declining and stationary trends in the caries prevalence and severity amongst children.^[5]^

Many individual risk factors such as poor oral health behavior, lack of knowledge and skills, and unhealthy diet play an important role on developing caries.^[6]^ International and macro-level factors promote the development of caries through alterations to the living environment and food choice. A number of comprehensive frameworks have shown the pathways through which international and macro-level factors such as globalization, development, and media programs and advertisement affect health outcomes positively and/or negatively.^[7,8]^ A modified framework model has been suggested for the way abovementioned international factors relate to caries.^[6]^ Researchers have focused on the pathways that link globalization with general health. These frameworks are ranging from a complex and detailed one,^[9]^ to mid-level complexity and simplified frameworks.^[10,11]^

Globalization by its definition encompasses greater integration within the world economy through movements of goods and services and also involves the spread of knowledge, science and culture with increase of the global interconnections.^[8,12]^ Therefore, the process of globalization has led to nutritional and lifestyle transitions, resulting diseases, and health services by its impact on national income and the trade.^[13,14]^ Although it is difficult to directly link nutrition transition with globalization, macro-level factors that lead to changes in diet and nutrition within social systems are linked to globalization such as urbanization, economic growth, free trade liberalization,^[11,15]^ education.^[7,8]^

According to the Swiss Economic Institute, Konjunkturforschungsstelle Globalization Index (KOFGI) is the most profoundly adopted and cited index in the literature.^[16]^ This index covers three main dimensions of globalization: economic, social and political. Variable such as international treaties and number of partners in investment treaties are used to measure the political dimension of globalization. Economic freedom involves removing barriers for free trade and foreign investment,^[17]^ including investment in the distribution of food and through spreading out of fast-food chains and multinational food companies.^[18]^ The liberalization of international food trade and foreign direct investment and growth of transnational food corporations, beside global food advertising and promotion, all resulted in increased availability of processed foods, and increase in desirability, diversity, and price of food, which eventually led to nutritional transition.^[11]^ KOFGI measures the economical dimension of globalization using trade and foreign direct investment as a percentage of gross domestic product (GDP).^[16]^ These two variables have a causal relationship with economic growth.^[19]^ Economic growth leads to increase in the trade of goods such as animal products, refined grains and sugar.^[15]^ Hence, rapidly developing countries go through nutrition transition and change on lifestyle at initial stages of economic growth and development.^[20]^ Cultural globalization is one of the measures for the social dimension.^[16]^ The index uses variables such as the number of McDonald's restaurants and IKEA stores in the country to measure cultural globalization. Globally, more people live in urban areas than in rural areas, with 55% of the world's population residing in urban areas in 2018. In 1950, 30% of the world's population was urban, and by 2050, 68% of the world's population is projected to be urban.^[21]^ This global urban growth might lead the McDonald's, IKEA and other brands to spread out and increase in number. Urbanization has led to changes in the living environment and lifestyle, besides the availability of a range of food choices in which all have a direct influence on the quality of diet and energy expenditure.^[15]^ In this study we hypothesize that globalization is related with caries prevalence through the macro-level factors; economic freedom, economic growth and urbanization, as these factors lead to nutritional transitions that ultimately affect caries via biological pathways. The present study aimed to identify the global trend of dental caries according to level of national income, and to examine the role of globalization, health services expenditures, obesity, and sugar consumption on caries prevalence.

## 2 Materials and methods

### 2.1 Data source

**Figure 1:**
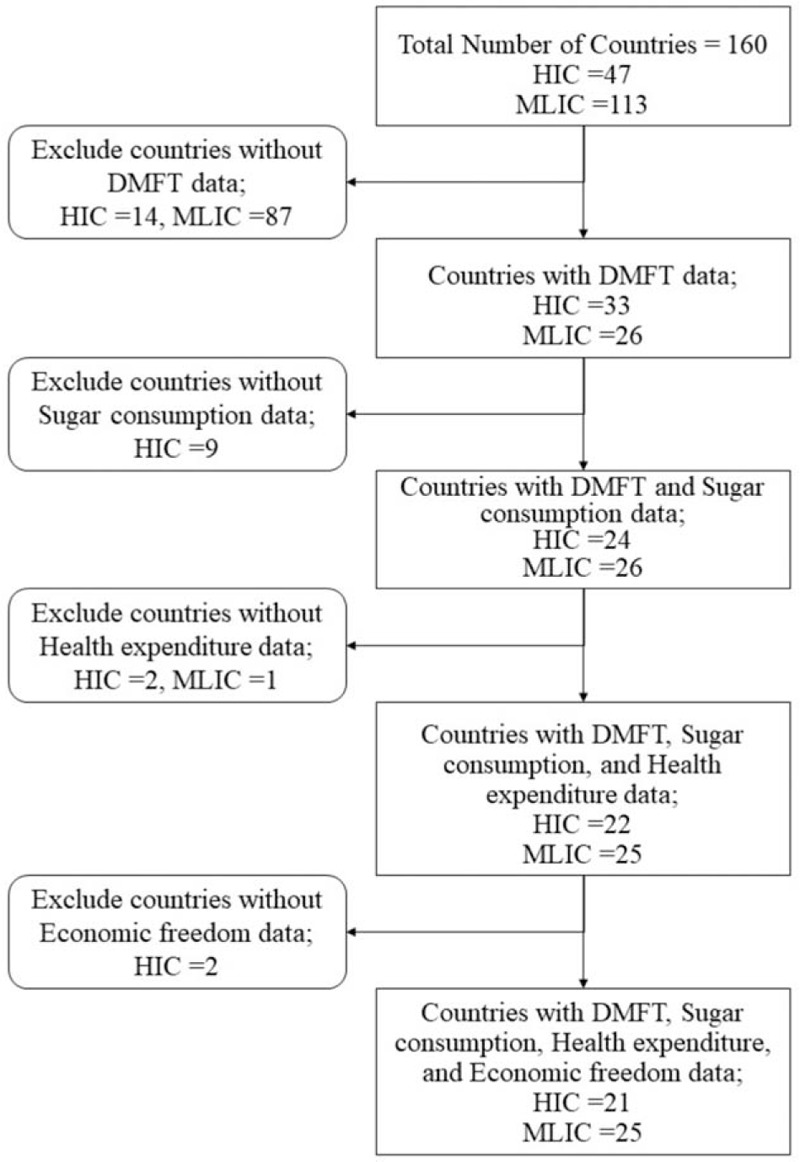
Flow summary of countries selection method. DMFT = decayed, missing, and filled teeth, HIC = high income countries, MLIC = middle and low income countries.

Data of dental caries, globalization, health service and health related factors for a total number of 160 countries were collected for the time period of 1990s to 2010s. Therefore, the years 1991, 2000 and 2012 were selected as representative years with an average of 10-year interval. The year 1991 was a representative of the late 1980s to the mid-1990s; the year 2000 denoted mid-1990s to mid-2000s, while the year 2012 represented the late 2000s and early 2010s, so the data for the adjacent year was used in case of the data was not available for the exact selected year. After that, the countries were divided according to their level of income based on World Bank classification for the year 2010 into two groups, the first group included High Income Countries (HIC) = 47, while the second group included Middle and Low Income Countries (MLIC) = 113.^[22]^ The final number of countries included in the study with complete data was 46, among them HIC = 21 and MLIC = 25. Figure 1 shows the flow summary of the selection method of countries.

The data were obtained from the online data bases of the following, Malmö University data of DMFT and sugar consumption^[23]^; Organization for Economic Cooperation and Development for countries with missing data of DMFT^[24]^; The World Bank data of GDP per capita based on purchasing power parity, health expenditure per capita (constant 2011 international $), public health expenditure as a percentage of GDP, and urban population % of total population^[25]^; Institute for Health Metrics and Evaluation-University of Washington data of obesity, mean value for the age group 10 to 14 years^[26]^; and the Heritage Foundation data of economic freedom.^[17]^ The approval of institutional review board was not necessary because all data used in this study were publicly open data.

### 2.2 Variables

The dependent variable was dental caries. It was represented by DMFT index values for 12-year-olds, which describe the caries experience in an individual.^[6]^ Globalization; the main independent variable, was measured by GDP per capita as an indicator for economic growth, urban population as an indicator for urbanization,^[15]^ and economic freedom. Health service and Health related Factors (HRF) were both mediator factors and served as dependent and independent variables. Health service was measured by health expenditure per capita and public health expenditure (% of GDP), while HRF was measured by sugar consumption and obesity. Eventually, time-variable is represented by the years 1991, 2000, and 2012.

Health service was represented by public health expenditure as a percentage of GDP that reflects government spending on health sector and also represented by health expenditure per capita that shows the peoples’ share on the spending on health services which in turn affects their use of these services and their general and dental health. Sugar consumption and obesity share common risk factors, and both have been associated with caries. Increase sugar consumption is one of the outcomes of globalization and there is evidence supports the relationship between the amount of sugars consumed and caries development.^[27]^ Obesity as well has been associated with caries and both share common risk factors.^[28]^

### 2.3 Statistical analysis

A one-way repeated measure analysis of variance (ANOVA) was first applied to compare the mean DMFT values of HIC and MLIC, and to compare these values within each group at the selected time periods. Mauchly's Test of Sphericity indicated that the assumption of sphericity had been violated (*P* < .05), therefore the Huynh-Feldt corrected tests are reported. Next, using multiple linear regression, the variables were adjusted in a serial fashion. The first model included sugar consumption and time. Both variables were chosen as the basic model because the association between sugar consumption and caries is already proved with evidence and the purpose was to see how other variables affect this association. Model 2 comprised of model 1 and obesity. In model 3, health service indicators were added to model 2. Lastly, in model 4, globalization indicators were added to the other variables in model 3. Multiple linear was carried out using SPSS.23.0 (IBM Corp. Armonk, New York) and the critical level of statistical significance was set at *P* < .05.

Finally, for the complicated relationships between variables, Partial Least Squares Structural Equation Modeling (PLS-SEM) was conducted. PLS-SEM is a powerful analytical method because it works competently with small sample size, can be applied to any data scale, and no distributional assumptions are needed.^[29]^ Three conceptual variables were constructed; health service was constructed by health expenditure per capita and public health expenditure, HRF by sugar consumption and obesity, and globalization by economic freedom, GDP per capita, and urban population. The research models were built up hypothesizing that with time, globalization affects dental caries directly and indirectly via health services and HRF. Hypothesis testing was completed by calculating β and *P*-values of the path models with *P*-value less than .05 was considered statistically significant. We did not investigate the path from health service towards HRF as there is no evidence for the relation between them. Time has been added to the model to examine its effect on caries and compare it with the results of the multiple linear regression.

The measurement models assessment for both groups; HIC and MLIC, are shown in Table 1. All constructs in the models fulfilled the requirement for reliability except for HRF in the HIC model.^[30]^ Convergent validity was assured by the assessment of the outer loadings. All outer loadings were significant (*P* < .05) except HRF indicators in HIC model, and all exceeded the minimum threshold of 0.4 for exploratory research as suggested by the study of Hulland.^[31]^ The average variances extracted (AVE) of the constructs in the proposed models were all more than adequate; over 0.5, except for HRF in the HIC model.^[32]^ For the discriminant validity assessment, all indicators had the highest loading in their corresponding construct in the model and the square roots of AVEs of each construct were greater than the correlation of the specific construct with any of the other constructs in the model except for globalization as it was highest with HRF in the MLIC model.^[33]^

**Table 1:**
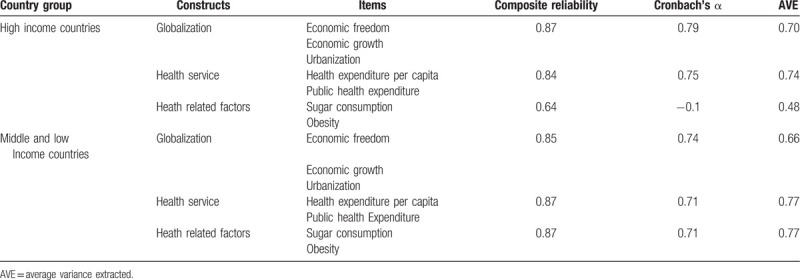
Results of measurement models assessment.

Hypothesis testing was done by the assessment of structural models. A bootstrapping technique with 500 adjusted samples was applied to check the validity of the structural relationships among latent variables and to estimate the significance of the path coefficient. The exploratory path models that were tested in this study demonstrated the hypothesized relationships between the independent latent variables and caries. The results of the preliminary collinearity check showed that all variance inflation factors (VIFs) below 5.0. The *f*^2^ effect size of each path for HIC model reveals that the paths from globalization to dental caries and health service had large effect size. On the other hand; the *f*^2^ effect size of each path for MLIC model shows that the paths from globalization to health service and HRF had large effect size. Whereas the path from time to caries had medium effect size.^[30]^

The explanatory power with *R*^2^ values (adjusted *R*^2^) revealed that in HIC model, the independent latent variables explained 52.6% of the variance in dental caries, which indicative of a very strong explanatory power.^[33]^ On the other hand, MLIC model explained 23.8% of the variance in caries, which revealed a moderate explanatory power. Furthermore, both models have acceptable predicative relevance with over zero *Q*^2^ value.^[32]^ SmartPLS version 3.0 statistical package is used for the assessment of data analysis.

## 3 Results

**Figure 2:**
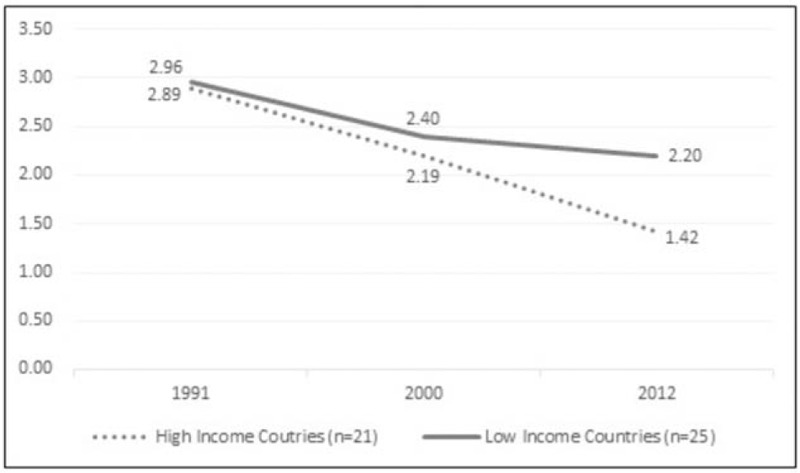
Dental caries trend in high vs middle and low income countries.

The trend of caries showing a continuous decrease in DMFT in HIC, while in MLIC, there is a slight decrease in the DMFT after the new millennium comparing to a more former strident decline (Fig. 2). The results showed that there was no significant interaction between the two groups at all time periods as both groups showed a declined DMFT trend (*F*(1.85, 81.5) = 1.95, *P* = .152). However, there was a significant difference between groups in the mean DMFT index only in the year 2012 (*P* < .01). The mean DMFT index was always higher in MLIC than HIC at each time period. Moreover, the mean difference was significant for all within group comparisons (*P* < .05), except for the years 2000 and 2012 in MLIC.

The results of the multiple linear regression for both groups of countries; HIC and MLIC, are shown in Table 2. In model 1, sugar consumption had a positive and significant association in MLIC. In model 2, obesity had a trivial effect on the correlations in model 1 in both groups. After adding health service indicators; model 3, the association between sugar consumption and caries became stronger and statistically significant in HIC. The final model included all the variables after the addition of economic freedom, GDP per capita, and urban population to those in model 3. The correlation between sugar consumption and caries became weaker and non-significant in both groups of countries.

**Table 2:**
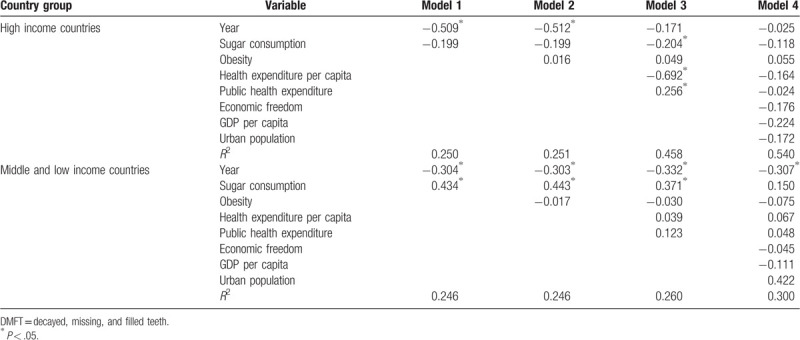
Regression models for the association between different variables and dental caries (DMFT) represented by β coefficient.

**Figure 3:**
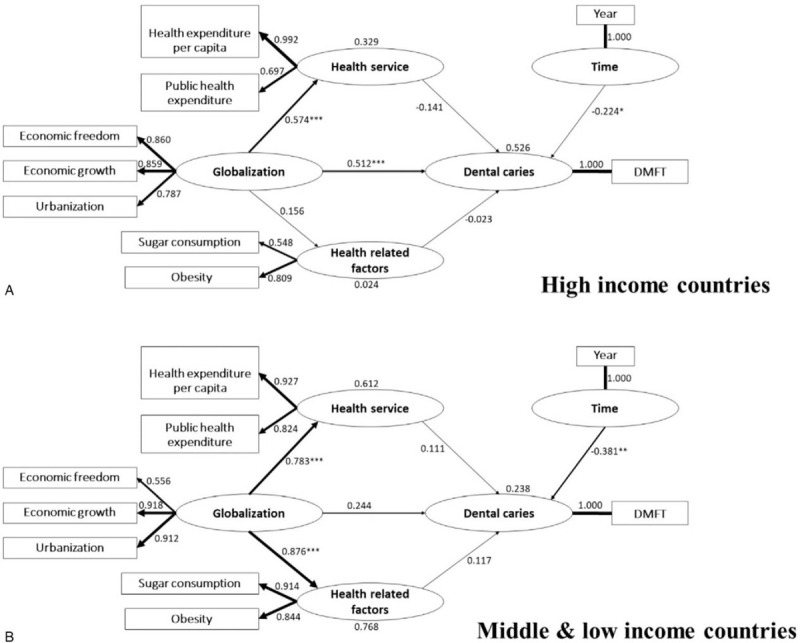
Path diagrams showing the path coefficient. (A) High income countries. (b) Middle and low income countries. Significance level was set at ^∗^*P* < .05; ^∗∗^*P* < .01; ^∗∗∗^*P* < .001.

Turning to the results of PLS-SEM, Figure 3 shows the pathways parameters for the HIC model and the MLIC model respectively. On the contrary to the results of the multiple linear regression, conceptual variable “globalization” had a strong negative and significant association with caries (β = –512, *P* < .001) in HIC, meaning that globalization has an effect on caries decreasing trend when countries become more globalized. Similar to the results of the multiple linear regression, time had a strong negative and significant correlation with dental heath in HIC and MLIC (β = –0.224, *P* < .05) (β = –0.381, *P* < .01) respectively. Globalization had a strong positive and significant association with health service (β = 0.574, *P* < .001) in HIC; implying that countries are more likely to provide a better health service as they become more globalized. In MLIC, globalization had a very strong positive and significant correlation with both health service and HRF (β = 0.783, *P* < .001) (β = 0.876, *P* < .001) respectively, denoting that a gain provision of health service may have improved as countries become more globalized. Additionally, HRF in terms of sugar consumption and obesity increase with globalization.

## 4 Discussion

This study presented that caries trend for HIC and MLIC showed continuing declination, and this go in line with previous studies.^[5,34]^ However, the declined pattern of caries trend has been attenuated in MLIC after the new millennium indicating that an event has led to this change. Then, this study explored the time effect of globalization, health service and HRF on caries as the hypothesized explanation for the change of caries trend in MLIC.

The results of the multiple linear regression showed that health service (health expenditure and public health expenditure) had a greater impact on the association between sugar consumption and caries in HIC than MLIC. This difference could be referred to the higher expenditures for health service, and that a high percentage of these expenditures being directed towards dental health in HIC more than MLIC. These expenditures are invested in treatment and preventive oral care in HIC and whereas in MLIC are mostly used for emergency oral care and pain relief.^[35,36]^ The results also showed that individual globalization indicators (economic freedom, GDP per capita, and urban population) had no dramatic role on caries trend. The linear regression cannot show the role of individual globalization indicators on the change of population dental health. This is because it cannot account for the complicated relationships among different variables, as it shows the individual effect of these variables not a combined one. Since then, PLS-SEM was used as it offers a great potential to test relationships as a comprehensive means.

The results of PLS-SEM showed that the comprehensive variable “globalization” constructed by economic freedom, GDP per capita, and urban population had a great positive association with caries reduction in HIC; however, it had a weak influence in MLIC. This is might be due to the availability and affordability of fluoridated toothpaste and preventive products in HIC, which make them widely used. Beside the high GDP for these countries, which increases the household income that leads to better use of these products and health services. However, this is not the case in MLIC in which these products are not commonly used,^[37]^ alongside the low household income, which results in lower use of health services.

Globalization has led to a high increase of health expenditure in both groups of countries especially MLIC as a result of economic growth and the increase of GDP. In relation with HRF, globalization had a very strong influence on the increase of the rate of sugar consumption and obesity in MLIC. As economic freedom and policies of trade liberalization lead to raise the availability of sugar and processed food with low nutritional value, which associated with increased rates of obesity and other non-communicable diseases.^[38,39]^

The results of PLS-SEM also showed that the association between health service and caries prevalence in HIC was negative but not significant. This might be due to that the DMFT scores were represent the F component of the index (demonstrates treated caries) which is an indication of greater access to dental services in developed countries. Another possible explanation is that dental caries is higher among the lower socio-economic class who has inadequate access to dental services in HIC.^[34]^ The PLS-SEM result of the relation between health service and caries in HIC was different from those of the multiple linear regression. Further research on the actual relation between health service and caries is needed. On the other hand, health service had a positive association with caries in MLIC. This might be due to the share of dental health expenditure from the total health expenditure is low in MLIC and mostly used for emergency oral care and pain relief. While in HIC the dental health expenditure is higher than in MLIC, and these expenditures are invested in treatment and preventive oral care.^[35,36]^ HRF had contrasting associations with caries as they had negative association in HIC but positive one in MLIC. This could be due to inadequate exposure to fluorides and absence or limited public health measures with the increase of sugar consumption and processed food in MLIC.^[35]^

Our study has several inevitable limitations that should be considered: first, the objectives of this study were assessed on an ecological level. Therefore, the study is subjective to some methodological limitations mainly “ecological fallacy”. Besides that, the difficulties in ensuring the reliability and validity of the measures, particularly given the range of different groups involved in the data collection,^[40]^ mainly on caries prevalence. Additionally, the data were often collected at different time points, which may have introduced some degree of variation in the measurement. Second, due to the repeated cross-sectional design study design, only limited convincing conclusions could be drawn from this study. Third, although this study highlights important associations, it does not demonstrate causality. Finally, globalization may have a role on dental health through oral health behavior, oral health programs and education, but we cannot test this role due to lack of data, so that could be a subject for a future research. In case of PLS-SEM, all items were maintained in the models and that is because of: (1) this is an exploratory research, (2) used for comparison propose between two groups and (3) that the data used were secondary data. Our study does, however, have the following advantages. It is the first study to explore the role of globalization on the global caries trend in a comparison between HIC and MLIC using different statistical methods. Besides that, we used all the available online data of the selected variables.

In conclusion, caries trend in HIC and MLIC has shown a declination pattern over time but this pattern has been attenuated after the new millennium in MLIC. From our findings it can be implied that the globalization was associated with lower DMFT in HIC, and had a strong association with increase of the health expenditure in both groups of countries especially MLIC. Moreover, globalization has been associated with increase of sugar consumption and obesity in MLIC. Therefore, based on these findings, this study suggests that regulations and policy change related to the trade of sugar has to be implemented, particularly in MLIC as they considered the favorite market for big food companies. Future research is to be directed to examine the role of oral health behavior, measures and educational programs, and type of health and political systems on the relation between globalization and dental health.

## Acknowledgments

We are grateful to Professor *Jihyun Lee* and *Changyup Kim* for their support in implementing this research.

## Author contributions

Dong-Hun Han designed the study. Bakr Salem Alsuraim collected the data, performed the analysis, and drafted the manuscript. All authors read the manuscript and are responsible for it.
